# Molecular alterations in the *GATA-2*, *RUNX1*, *C/EBPα* and *hTERT* genes in patients with aplastic anemia by MLPA

**DOI:** 10.1590/1678-4685-GMB-2025-0127

**Published:** 2026-05-15

**Authors:** Iveth Mendoza Salas, Irma Olarte Carrillo, Rafael Cerón Maldonado, Anel Iraís García Laguna, Adrián De la Cruz Rosas, Christian Omar Ramos Peñafiel, Efreen Horacio Montaño Figueroa, Diana Laura Carrillo Rocha, Carlos Martínez Murillo, Verónica Fabiola Morán Barroso, Adolfo Martínez Tovar

**Affiliations:** 1 Hospital General de México “Dr. Eduardo Liceaga”, Servicio de Hematología, Laboratorio de Hematología, Ciudad de México, Mexico.; 2 Hospital General de México “Dr. Eduardo Liceaga”, Servicio de Hematología, Ciudad de México, Mexico.; 3 Hospital General de México “Dr. Eduardo Liceaga”, Servicio de Genética, Laboratorio de Bioinformática, Ciudad de México, Mexico.

**Keywords:** MLPA, Aplastic Anemia, molecular alterations

## Abstract

Aplastic anemia (AA) is a disease characterized by a severe reduction of the erythroid lineage. Its molecular mechanisms have been studied using technologies such as whole-exome sequencing via NGS; however, this remains a costly and limited-access strategy for developing countries. In this study, 17 *de novo* patients diagnosed with AA were analyzed. Genomic DNA was isolated from each patient to perform the Multiplex Ligation-dependent Probe Amplification (MLPA) technique, which uses different probes to detect numerical alterations in the exons of the genes of interest (*GATA2*, *RUNX1*, *C/EBPα*, *hTERT*). In 70.9% cases, a molecular abnormality was found. *GATA2* was the most frequently altered gene (58.8%), followed by *TERT* (47.0%), *RUNX1* (41.1%), and finally *C/EBPα* (35.3%). Detecting these molecular alterations could help to understand the progression of AA to other hematologic malignancies due to the genomic instability associated with this panel of genes involved in various hematopoietic maturation processes.

## Introduction

Aplastic anemia (AA) results from multiple molecular and immunological factors that cause hematopoietic failure and suppression, and is characterized by pancytopenia (Schoettler and Nathan, 2018; Wang et al., 2023). It has been described that certain transcription factors such as GATA-2, RUNX1, C/EBPα, and those involved in telomere maintenance (hTERT) are implicated in hematopoiesis. However, it has not been reported whether these molecular alterations are present in aplastic anemia (Yoshizato et al., 2015).

The GATA-2 transcription factor is expressed in hematopoietic stem cells and early progenitors, playing a critical role in hematopoiesis (Xu et al., 2009; Vicente et al., 2012). In patients with AA decreased expression of GATA-2 has been reported, inhibiting proliferation and survival of hematopoietic stem cells (Harigae, 2006; Shimizu and Yamamoto, 2016). The *RUNX1* gene is a transcription factor that controls cell fate across various hematopoietic lineages (Krishnan, 2023), playing essential roles in many biological processes, including embryonic development, cell proliferation, differentiation, and apoptosis (Bae et al., 2019; Samarakkody et al., 2020).

RUNX1-deficient cells show defects in DNA repair mechanisms, and mutations in this gene may occur in 20% of Fanconi anemia cases (Sood et al., 2017). Another gene that may be involved in AA is *C/EBPα*, which is implicated in cell proliferation and differentiation (Friedman, 2015; Avellino and Delwel, 2017). Dysfunction in this gene causes an early and selective block in granulocyte maturation without affecting other hematopoietic lineages (Pabst and Mueller, 2009; Wang et al., 2012). However, its role in erythroid lineage maturation has not been reported. Finally, premature telomere shortening in patients with this condition causes bone marrow failure (Savage et al., 2006 a , b).

Reduced *hTERT* gene expression is associated with decreased enzyme activity and telomere maintenance (Ball et al., 1998). Molecular alterations in this gene impact prognosis in patients with aplastic anemia, being associated with higher relapse risk, lower overall survival, and risk of clonal evolution (Shallis et al., 2018). 

It is necessary to alert about the precise molecular diagnosis of aplastic anemia and to distinguish between MDS and congenital diseases, so studying the molecular alterations of a group of genes is of great relevance in this work.

Therefore, the aim of this study was to identify alterations in the *GATA-2*, *RUNX1*, *C/EBPα*, and *hTERT* genes using the Multiplex Ligation-dependent Probe Amplification (MLPA) technique in patients with aplastic anemia and evaluate their possible prognostic significance, through a fast and accessible methodological strategy.

## Subjects and Methods

### Study population

Seventeen adult patients (12 men and 5 women) from the hematology department of the Hospital General de México Dr. Eduardo Liceaga were analyzed. Bone marrow samples were obtained with prior informed consent. A fraction of the aspirate collected for diagnostic purposes was sent to the research laboratory for analysis.

### Leukocyte isolation

Heparinized bone marrow samples were collected with informed consent from patients with aplastic anemia. Erythrocyte lysis solution (Roche Applied Science) was used to isolate leukocytes. The obtained leukocytes were stored at -80°C until DNA extraction.

### DNA isolation

Genomic DNA was isolated using the DNAzol^®^ reagent (Life Technologies). During isolation, leukocytes were lysed with DNAzol following the manufacturer’s instructions. The isolated DNA was stored at -20°C until use.

### Multiplex Ligation-dependent Probe Amplification (MLPA)

MLPA was performed using the SALSA^®^ MLPA^®^ Probemix P437-B1 kit, Lot A01, according to the manufacturer’s instructions (www.mrc-holland.com).Genomic DNA was diluted and denatured, followed by hybridization with the SALSA^®^ MLPA^®^ Probemix P437-B1 Familial MDS-AML Lot A01 probes. Ligation was then performed, followed by PCR amplification. After PCR, fragments were analyzed via electrophoresis using the ABI PRISM^®^ 3100 Genetic Analyzer (Applied Biosystems) and the LIZ GS 500 size marker. The resulting fragments were analyzed using Coffalyser V 1.0 software (MRC-Holland). Deletions and duplications in the *GATA-2, RUNX1*, *C/EBPα*, and *hTERT* genes were determined according to the Relative Peak Ratio (RPR) of the electropherogram, with a normal range of 0.7-1.3.

### Statistical analysis

A risk analysis was performed to evaluate the impact of clinical and genetic variables on patient response. A p-value of less than 0.05 was considered statistically significant. All statistical analyses were performed using SPSS version 25 (IBM, Armonk, NY, USA). 

### Ethical considerations

This trial is a minimum risk investigation and was conducted based on the Declaration of Helsinki. For its realization, it was approved by the Ethics and Research Committees of the General Hospital of Mexico “Dr. Eduardo Liceaga”, with registration number DI/16/103/03/035.

## Results

Seventeen patients diagnosed with aplastic anemia were analyzed, with a predominance of males (70.5%) over females (29.4%). The average age was 42 years. According to Camitta’s criteria, most patients presented with very severe anemia (58.3%), severe (11.7%), and non-severe (29.4%). Additionally, 64.7% of the patients showed a partial response, while 35.3% did not respond (Table 1). MLPA analysis (Table 2) revealed that 12 patients (70.5%) had at least one numerical molecular alteration (deletion or duplication) affecting one or more of the following genes: *GATA-2*, *RUNX1*, *C/EBPα*, and *hTERT*. The remaining 5 patients (29.5%) had no detectable abnormalities.

**Table 1 -:** Clinic characteristics of aplastic anemia patients.

Clinical parameters	N=17
Sex	
Male	70.58% (12/17)
Female	29.41% (5/17)
Age (y)	
Media	42.29 ± 18.11
Min	21
Max	74
Hemoglobin (g/dL)	2.01 ± 6.98
Leukocytes (x10^3^/μL)	2.04 ± 0.96
Neutrophiles (x10^3^/μL)	0.90 ± 0.74
Platelets (x10^3^/μL)	14.88 ± 10.90
Camitta criteria	
Non severe	29.41% (10/17)
Severe	11.76% (2/17)
Very severe	58.82% (5/17)
Response	
No response	35,3% (6/17)
Partial	64,7% (11/17)
Complete	-

**Table 2 -:** Copy number changes and mutations identified by MLPA in patients with aplastic anemia.

CASE NO.	*GATA-2*	*hTERT*	*C/EBPA*α	*RUNX1*	*TERC*	RESPONSE
1	Dup intr 1	NA	Dup 1	NA	NA	No response
2	NA	NA	NA	NA	NA	No response
3	NA	NA	NA	NA	NA	No response
4	Del intr 1 Del 1 Mut R398W	Del 1	Del 1	Dup 3	NA	No response
5	Del 1,3,4, 5 Del intr 1 Mut R398W	Del 1, 5, 16	Del 1	Del 9 Dup 3	NA	Partial
6	NA	NA	NA	NA	NA	Partial
7	Dup 8	NA	NA	NA	NA	Partial
8	NA	NA	NA	NA	NA	Partial
9	Mut R398W	Del 1	NA	Dup 3	NA	Partial
10	NA	NA	NA	NA	NA	Partial
11	Del intr 1 Del 1 Mut R398W	NA	Del 1	NA	NA	Partial
12	NA	Dup 14	NA	Dup 3,7	NA	No response
13	Del intr 1 Del 1,3 Del 4	Del 1	Del 1	Dup 3	NA	Partial
14	NA	NA	NA	NA	Del 1	Partial
15	Dup 4 Dup 8 Mut R398W	Dup 3,4,6,8,10, 11,13,14	Dup 1	NA	NA	Partial
16	Del intr 1 Del 1	Del 1	Del 1	Dup 3	NA	No response
17	Mut R398W	Dup 8, 13, 14	NA	NA	NA	Partial

Regarding the *GATA-2* gene, alterations were observed in 10 patients (58.8%). The main alterations found were heterozygous deletions in intron 1 (5/17, 29.4%) with an RPR of 0.45, followed by heterozygous deletions in exon 1 (5/17, 29.4%), exon 3 (2/17, 11%), and exon 4 (2/17, 11%) with RPRs of 0.32, 0.24, and 0.55, respectively. Only one patient showed a heterozygous duplication in intron 1 and exon 4, and two patients in exon 8, with RPRs of 1.34, 1.43, and 1.55. Regarding the frequency of the R398W mutation in GATA-2, it was found in 6 patients (35%).

For the *RUNX1* gene, alterations were reported in 7 patients (41.1%). The most frequent alteration was a heterozygous duplication in exon 3 (6/17, 35%), followed by one case in exon 7 (1/17, 6%), with RPRs of 1.55 and 1.66, respectively. Only one patient (6%) showed a deletion in exon 9 with an RPR of 0.45.

Alterations in the *C/EBPα* gene were found in 7 patients (41.1%). The most common was a heterozygous deletion in exon 1 (5/17, 29.4%) with an RPR of 0.56, and heterozygous duplication in exon 1 in 2/17 (11.7%) with an RPR of 1.46.

As for the *hTERT* gene, 8 patients (47%) had alterations in at least one exon, mainly heterozygous deletions in exon 1 (5/17, 27.5%) with an RPR of 0.57, followed by heterozygous duplications in several exons in 3 of them (17.6%). Three patients (17.6%) showed heterozygous duplications in exon 14 (RPR 1.46), and two patients (11.7%) had duplications in exons 8 and 13, with RPRs of 1.54 and 1.65, respectively.

**Figure 1 -:**
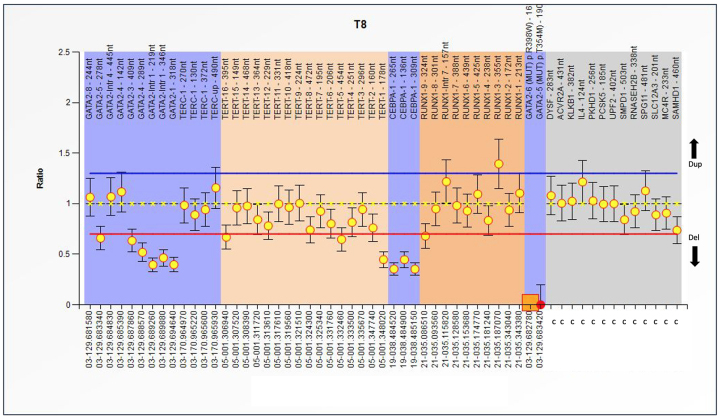
MLPA analysis of the Familial MDS-AML (SALSA^®^MLPA^®^Probemix P437). Abscissa represents several genes and control probes; ordinate represents fluorescent intensity of amplification. For each probe, the ratio <0.70 stands for deletion; and the ratio >1.3 stands for duplication. For this patient, *GATA-2*, *hTERT* and *C/EBPAa* presents deletion, duplication only for *RUNX1* in exon 3. The mutation R398W for *GATA2* is also present. For this patient the CAMMITA criteria is non-severe.

**Figure 2 -:**
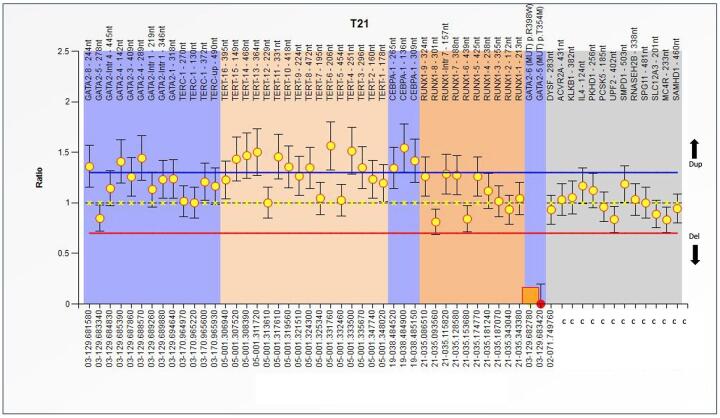
MLPA analysis of the Familial MDS-AML (SALSA^®^MLPA^®^Probemix P437). Abscissa represents several genes and control probes; ordinate represents fluorescent intensity of amplification. The ratio <0.70 stands for deletion; and the ratio >1.3 stands for duplication.

**Figure 3 -:**
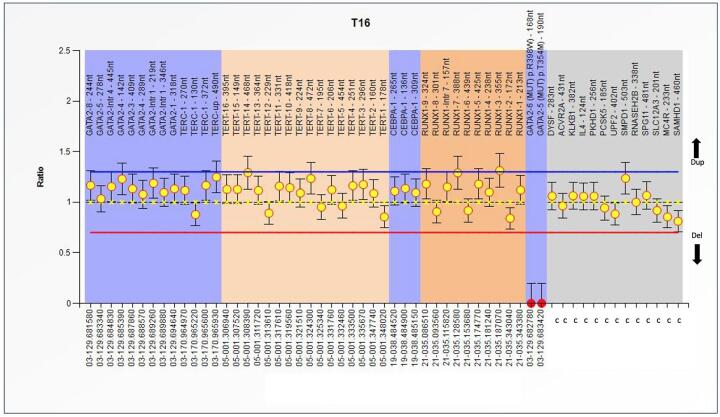
MLPA analysis of the Familial MDS-AML (SALSA^®^MLPA^®^Probemix P437). Abscissa represents several genes and control probes; ordinate represents fluorescent intensity of amplification. The ratio <0.70 stands for deletion; and the ratio >1.3 stands for duplication. Represents a patient only with duplications in several exons of *hTERT*, *GATA-2* and all the sites of *C/BPAa*. The mutation R398W for *GATA-2* is also present.

When analyzing alterations by patient, we found that 4 out of 17 patients (23%) presented alterations in the *GATA-2*, *hTERT*, *C/EBPα*, and *RUNX1* genes. Two of these patients did not respond to treatment, while the others showed a partial response. Finally, only 2 out of 17 patients (11.7%) had a single alteration with a partial treatment response. Patients with the R298W mutation in *GATA-2* also had heterozygous duplications in more than two *hTERT* exons Figure 1-3.

**Figure 4 -:**
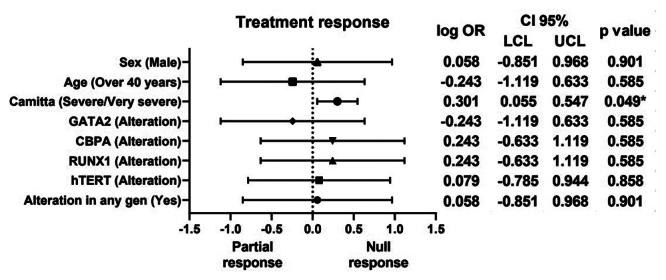
Association of odds ratio between clinical features/gene alteration and response to treatment in patients with aplastic anemia.

When evaluating the presence of genetic alterations, no significant results were found for the genes *hTERT* (OR 1.2, CI 0.164 - 8.799, p=0.858), *RUNX1* (OR 1.75, CI 0.233 - 13.159, p=0.585), *C/EBPα* (OR 1.75, CI 0.233 - 13.159, p=0.585), *GATA-2* (OR 0.571, CI 0.076 - 4.297, p=0.585), observing that the only variable that had an impact on treatment response was the risk criterion Camitta (OR 2.0, CI 1.136 - 3.522, p=0.049), confirming that patients with severe or very severe aplastic anemia are at greater risk of not achieving any type of response to treatment (Figure 4).

## Discussion

Aplastic anemia is characterized by a decrease in red cell lines. Immune system failure is one of the main causes of pancytopenia; however, other molecular alterations may also contribute, leading to defects in erythropoiesis. These include genes involved in hematopoiesis (*GATA-2*, *RUNX1*, *C/EBPα*), and genes encoding the telomerase enzyme (*hTERT*). Current treatment focuses on immune suppression using eltrombopag and cyclosporine, but targeted therapies could also be considered. 

In this study, we analyzed 17 plastic anemia patients with varying degrees of severity. They received treatment with cyclosporine, Danazol, and anti-thymocyte globulin, which form part of the institutional protocol. Using MLPA to detect molecular alterations, we found deletions and duplications in 70.5% of patients. It has been reported that approximately 75% of AA cases are idiopathic, 15% are due to drug use or infections, and only 5-10% are related to somatic abnormalities (Wang and Liu, 2019). Until now, no study has analyzed alterations in these genes in AA using MLPA, although it is widely used in other diseases.

We found that 58.8% of patients presented alterations in *GATA-2*. The gene plays a key role in hematopoiesis, erythrocyte and megakaryocyte development, and the maintenance of hematopoietic stem and multipotent progenitor cells (Viger et al., 2008). We observed heterozygous deletions in intron 1 and exons 1, 3, and 4.

These alterations affect the function of the protein, a zinc finger DNA-binding transcription factor, possibly disrupting the activation of target genes by interfering with consensus sequence binding (Wlodarski et al., 2017). Patients with GATA-2 deficiency frequently present with hypocellular bone marrow (BM) and features of OM insufficiency (McReynolds et al., 2018) with progressive loss of cell lineages over time. (Dickinson et al., 2014) 

The encoded protein plays an essential role in regulating the transcription of genes involved in the development and proliferation of hematopoietic and endocrine cell lineages (Pihlajoki et al., 2016). The duplications found in intron 1, exons 4 and 8 affect the C-terminal zinc finger region, which is crucial for DNA binding and consensus sequence recognition (A/T)GATA(A/G), providing protein stability (Peters et al., 2023). GATA-2 deletions are linked to myelodysplastic syndromes and acute myeloid leukemia (Bresnick et al., 2020). Another reported GATA-2 abnormality is reduced mRNA expression in AA patients versus healthy subjects, as well as in CD34+ bone marrow cells, suggesting that decreased expression may contribute to AA pathogenesis (Zhang et al., 1999). Thus, identifying these molecular alterations (deletions, duplications, mutations, and mRNA levels) may be relevant to the pathogenesis of this disease and its potential progression to MDS and AML.

In a study by McReynolds et al. (2018), molecular alterations in AA patients from three specialized centers were analyzed using whole-exome sequencing (NGS), detecting mutations and SNPs in genes like *BCOR*, *PIGA*, *DNMT3*, *ASXL1*, *JAKs*, *TP53*, among others, but no mutations or SNPs in *GATA-2* (Yoshizato et al., 2015). 

The *GATA-2* mutation, R398W, located in the exon 6, c.1192C>T (p.Arg398Trp) is considered as highly pathogenic, with monocytopenia with susceptibility to infections as well as provoke greater susceptibility to acute myeloid leukemia.

Our results showed that the mutation was found in the35.3% of patients, affecting the zinc finger domain, impairing DNA binding and resulting in defective hematopoiesis as mentioned above and according to the database consulted, ClinVar, COSMIC or dbSNP (Hahn et al., 2011; Bresnick et al., 2020).

In general, there are no reports that address the alterations of the *GATA-2* gene in this type of condition, the MLPA study allows us to know some alterations in the *GATA-2* gene, however we are aware that the number of samples must be increased and multicenter studies must be carried out.

Additionally, 35.2% of patients had molecular alterations in *RUNX1*. We found duplications in exons 3 and 7, and a deletion in exon 9 in a single patient. These regions are involved in lineage differentiation, maturation, and suppressor activity (Hong et al., 2019), *RUNX1* encodes a nuclear transcription factor involved in cell cycle regulation, ribosome biogenesis, hematopoietic differentiation, and the activation of TP53 and TGFβ. *RUNX1* mutations also occur in approximately 20% of patients with Fanconi anemia and 64% of patients with congenital neutropenia (CKD) who develop MDS. (Quentin et al., 2011; Skokowa et al., 2014).*RUNX1* mutations in SMD are distributed throughout the gene and affect both functional domains. *RUNX1* mutations also occur in approximately 20% of patients with Fanconi anemia and 64% of patients with congenital neutropenia (CKD) who develop MDS. (Quentin *et al*., 2011; Skokowa *et al*., 2014). *RUNX1* mutations in MDS are distributed throughout the gene and affect both functional domains. RUNX1 is one of the most frequently mutated genes in MDS, accounting for approximately 10% of cases. (Chen et al., 2007; Tsai et al., 2015).

In one AML study, *RUNX1* mutations were found in 115 patients (15%) via whole-exome sequencing (Guijarro et al., 2024). Notably, alterations in both *RUNX1* and *GATA-2* are considered drivers of hereditary hematopoietic malignancies (HHM) (Yi et al., 2022). Mutations in *GATA-2* carry a 90% risk of developing HHM, and 44% in *RUNX1*. Clonal hematopoiesis has been reported in abnormalities of both genes (Elagib et al., 2003).

We found that 41.1% of patients with AA had alterations in *C/EBPα*, with duplication in exon 1 being the most frequent. C/EBPα is a transcription factor containing a leucine zipper, involved in hematopoiesis, especially granulocyte maturation (Koschmieder et al., 2009). Alterations in this gene may cause a block in granulocytic maturation (Pulikkan et al., 2017). Deletions or duplications in the N-terminal domain can cause a reading frame shift, leading to a truncated 30 kDa protein and impaired cell maturation. Such alterations, especially mutations, occur in 5-14% of AML cases (Paz-Priel and Friedman, 2011). No reports exist on its alteration frequency in anemia.

Finally, 47% of AA patients had *hTERT* alterations. However, specific exon deletions or duplications in *hTERT* have not been widely reported. In a study (Peslak et al., 2017), mutations in *hTERT* were found via sequencin3g (Armanios, 2009), causing telomere shortening in AA patients versus healthy controls. Another study associated telomere shortening with PNH clones in AA, suggesting telomere degradation may contribute to MDS development (Yoshizato et al., 2015).

hTERT is crucial for telomere maintenance and cell survival. Telomerase is a ribonucleoprotein formed by a central dimer of reverse transcriptase (hTERT) and an RNA component (TERC), which serves as a template for RNA-dependent DNA synthesis (Ramlee et al., 2016). We found that 27% of patients had deletions in exon 1, which may impair reverse transcriptase activity and cause telomere shortening, as reported previously. Telomerase dysfunction is implicated in diseases such as dyskeratosis congenita, pulmonary fibrosis, aplastic anemia, and acute myeloid leukemia (Borssén et al., 2011). Most studies report mutations in various hTERT regions and accessory proteins essential for its function, as described by Yamaguchi et al., 2005, who analyzed 205 AA cases and reported mutations in hTERT, TERC, and related proteins.

## Conclusion

Identifying molecular alterations (duplications, deletions, and mutations) in aplastic anemia will help us understand the mechanisms of molecular dysregulation that lead to this disease, which may progress to the development of other hematological neoplasms due to the genomic instability accumulated from this panel of genes involved in various hematopoietic maturation processes. In our research center, where resources are limited and full-exome NGS technology is not readily available, MLPA is a viable alternative for addressing this type of pathology. These molecular alterations in AA may understand the molecular regulation mechanisms underlying this disease and its potential progression to other hematologic neoplasms due to the genomic instability associated with this panel of genes involved in various hematopoietic maturation processes.

## Data Availability 

 The data supporting the findings of this study are available upon request to the corresponding author.
